# Retraction: Digital twin–driven design and testing of laser shock processed aluminum–graphene composites for spacecraft docking tribology

**DOI:** 10.1371/journal.pone.0351991

**Published:** 2026-06-22

**Authors:** The PLOS One Editors

The *PLOS One* Editors retract this article [1] due to concerns about:

- Citation of numerous unretrievable references, including References 1, 2, 3, 4, 5, 6, 7, and 8
- Compliance with PLOS policies on Authorship and Artificial Intelligence Tools and Technologies

We regret that the issues were not identified prior to the article’s publication.

The first author declared that the citation errors arose due to issues pertaining to journal access, book reference DOIs, manuscript formatting using referencing software, and intelligence tools used to compile and format the reference list. The authors provided a revised reference list in their response; however, the Editors consider the errors to be too extensive to resolve with a correction.

All authors did not agree with the retraction.
